# Facile synthesis of carbon nanobranches towards cobalt ion sensing and high-performance micro-supercapacitors†

**DOI:** 10.1039/c9na00181f

**Published:** 2019-07-19

**Authors:** Qiao-Ling Chen, Xingjiang Wu, Hengyang Cheng, Qing Li, Su Chen

**Affiliations:** State Key Laboratory of Materials-Oriented Chemical Engineering, College of Chemical Engineering, Nanjing Tech University (former Nanjing University of Technology) Nanjing 210009 P. R. China chensu@njtech.edu.cn liqing1128@njtech.edu.cn +86-25-83172258 +86-25-83172258

## Abstract

We present a facile strategy for fabricating a new type of one-dimensional (1D) carbon nanomaterial named carbon nanobranches (CNBs) covered with botryoidal carbon dots (CDs) by direct pyrolysis of a green precursor (starch). The resultant CNBs display both photoluminescence and electrical conductivity and can be assembled into chemical sensors and energy-storage devices. In terms of their bright photoluminescence, CNBs with a fabulous crystalline structure are utilized as fluorescent probes to sensitively and selectively detect Co^2+^ with a very low detection limit of 2.85 nM and a wide linear concentration range from 10 nM to 1 mM. Moreover, an efficient micro-supercapacitor (micro-SC) is constructed based on conductive CNB fibers produced *via* a customized microfluidic spinning technique. The micro-SCs exhibit a large specific capacitance of 201.4 mF cm^−2^, an energy density of 4.5 μW h cm^−2^ and high cycling stability, and can successfully power 19 light-emitting diodes (LEDs). The main purpose of this paper is to offer a perspective into simplifying the connecting of research and industry by starting from green carbon-based materials.

## Abstract 2

A facile strategy is developed for fabrication of carbon nanobranches (CNBs) *via in situ* pyrolysis of starch.

## Graphical abstract

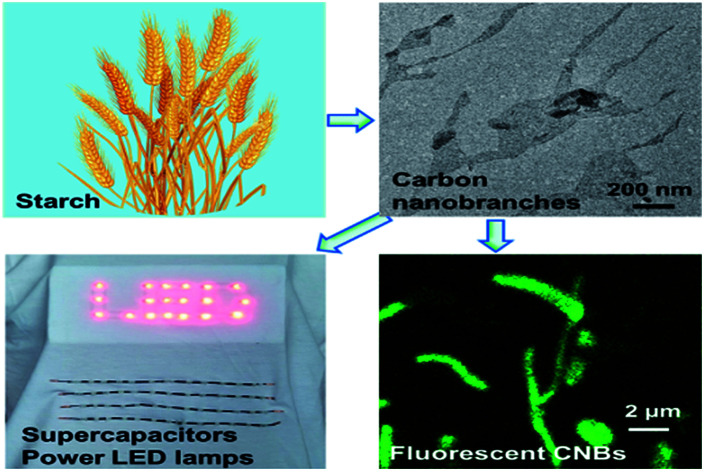

## Introduction

In the last few decades, 1D nanoscale carbon materials, such as nanotubes,^1,2^ nanofibers,^3–5^ nanowires,^6–8^ nanorods,^9,10^ and nanoribbons^11,12^ have attracted enormous interest among scientists either because of their unique properties in the thermal, mechanical, electrical, and optical fields, or their applications in field emission,^13^ nanoelectronics,^14,15^ sensors,^16,17^ solar cells,^18,19^ batteries,^20,21^ supercapacitors^22,23^ and hydrogen storage.^24,25^ Compared with wide investigation of carbon nanotubes and carbon nanofibers, other types of 1D carbon nanomaterials, such as carbon nanowires (CNWs), carbon nanorods (CNRs), graphene nanoribbons (GNBs) and carbon composite nanobranches,^26,27^ are drawing increasing attention due to their novel nanostructure and exceptional properties. Several new routes are proposed to prepare these materials, for instance, Meyer *et al.* developed linear atomic CNWs from graphene by utilizing a high-energy electron beam.^28^ Pachfule *et al.* reported a self-templated strategy to fabricate one-dimensional CNRs by carbonization of rod-shaped metal–organic frameworks at 1000 °C in an Ar flow.^29^ GNBs with sub-10 nm width were fabricated by employing chemically synthesized nanowires as the physical protection mask in oxygen plasma etching, and exhibited excellent room temperature transistor behavior with an on/off ratio around 160.^30^ Due to their unique structure, 1D carbon nanomaterials also serve as active sites by hybridizing with other materials to provide superior performance. The CNWs attached with uniform carbon quantum dots exhibited bright fluorescence by pyrolysis of highly aligned DNA nanofibers on poly(dimethylsiloxane) sheets.^31^ GNBs composited with SO_2_ were employed as anode materials for lithium-ion batteries, which improved electrochemical performances through high aspect ratio GNRs with their conductive paths.^32^ GNBs with graphene sheets can enhance the hydrogen evolution by a multi-pathway of charge and mass transport.^33^ In addition to all of this, researchers exploited 1D carbon nanomaterials in many other applications.^13–19^ However, there is almost no effective and facile approach to endow these 1D carbon nanomaterials with a brand new morphology and structure so as to exhibit multifunctional properties such as fluorescence and energy-storage ability.

Herein, we propose a facile approach for the construction of a new type of 1D carbon nanobranch (CNB) covered with abundant botryoidal carbon dots (CDs), showing both photoluminescence and electrical conductivity. The CNBs exhibit distinct advantages in their dual properties, compared with carbon nanowires with only photoluminescence,^31^ graphene nanoribbons with only electrochemical properties,^32^ and other 1D carbon materials with a single property.^14–23^ To the best of our knowledge, the dendritic structure of CNBs composed of botryoidal CDs is amazing and has never been reported so far, quite different from other reported nanobranches, such as silicon/carbon nanobranches,^26^ metal oxide nanobranches^34–36^ and metal nanobranches.^37,38^ In virtue of the excellent photoluminescence and electrical conductivity, CNBs were exploited for application in chemical sensors and micro-supercapacitors. Intriguingly, fluorescent CNBs exhibited superior selectivity and sensitivity towards cobalt ions. A detection limit as low as 2.85 nM can be reached along with a wide linear concentration range from 10 nM to 1 mM, suggesting excellent detection performance towards Co^2+^. Furthermore, highly conductive fibers composed of CNBs were synthesized *via* microfluidic spinning. Higher-performance fiber-based micro-supercapacitors (micro-SCs) were constructed to power LEDs successfully. Therefore, our method opens a new avenue to realize CNBs with not only a unique morphology and crystalline structure, but also higher fluorescence and energy-storage ability.

## Experimental

### Chemicals and materials

Reagent-grade potato starch, polyvinyl alcohol (PVA), and 4,7,10-trioxa-1,13-tridecanediamine (TTDDA) were purchased from Aldrich. Thermoplastic polyurethane (TPU) was supplied by Wanhua Chemical Group Co., Ltd. Reagent-grade *N*,*N*-dimethylformamide (DMF), dichloromethane (CH_2_Cl_2_), trichloromethane (CHCl_3_), acetone, tetrahydrofuran (THF), methanol, concentrated nitric acid (HNO_3_), and sodium carbonate (Na_2_CO_3_) were supplied by Sinopharm Chemical Reagent Co., Ltd. (Shanghai, China). Cadmium chloride hemipentahydrate (CdCl_2_·2.5H_2_O), cobalt acetate tetrahydrate (Co(Ac)_2_·4H_2_O), copper acetate monohydrate (Cu(Ac)_2_·H_2_O), magnesium chloride hexahydrate (MgCl_2_·6H_2_O), ferric chloride anhydrous (FeCl_3_), manganese chloride tetrahydrate (MnCl_2_·4H_2_O), nickel chloride hexahydrate (NiCl_2_·6H_2_O), lithium chloride monohydrate (LiCl H_2_O), zinc chloride anhydrous (ZnCl_2_), and ferrous chloride tetrahydrate (FeCl_2_·4H_2_O) were purchased from Sinopharm Chemical Reagent Co., Ltd. (Shanghai, China). Dialysis bags (molecular weight cut off = 14 000) were purchased from Shanghai Kayon Biological Technology Co., Ltd. Ultrapure water was used in all experiments.

### Fabrication of CNBs

Starch was used as the precursor in a pyrolysis process to produce carbon nanobranches. In a typical process, 1 g starch was mixed homogeneously with 1 mL, 2.0 wt% FeCl_3_ aqueous solution. The mixture was placed on a quartz boat and then the quartz boat was transferred into a quartz tube inside a tube furnace. Subsequently, the furnace was heated to 800 °C at 5 °C min^−1^ by a programmer. An Ar/H_2_ mixture was flowed into the reactor with a flow rate of 20 mL min^−1^ (Ar : H_2_ = 95 : 5) at a pressure of 0.1 MPa from beginning to end. The reaction temperature was maintained at 800 °C for at least 20 h. In order to remove Fe, the resulting sample was treated with concentrated hydrochloric acid using a reflux condenser for 6 h. The pre-CNBs were prepared. Then the pre-CNBs were dispersed in 30 mL of concentrated nitric acid at the reflux temperature for 24 h. The obtained brownish solution was neutralized with Na_2_CO_3_, and then dialyzed against ultrapure water with a cellulose ester membrane bag (*M*_w_ 14 000) to remove salts. Furthermore, 0.5 g of TTDDA was also added to the pre-CNB solution and the mixture was stirred and heated to 100 °C once again using a reflux condenser for up to 48 h with a flow of N_2_. The CNB solution was finally obtained by dialysis of the resulting solution once again to remove excess TTDDA. The supernatant of the CNB solution has abundant fluorescent CNBs. After evaporating water, the CNB powder was obtained.

### Fabrication of CNBs/TPU conductive fiber and fiber-shaped micro-SCs

CNBs/TPU conductive fibers are prepared as follows. 3 g TPU was added to 10 mL DMF solution with vigorous stirring to form a homogeneous solution. Then, 1 g of CNBs was added to the above solution, which was stirred at 60 °C for several hours. The conductive fibers were obtained *via* microfluidic spinning, followed by drying at 60 °C. 1 g H_3_PO_4_ and 1 mL deionized water were mixed together. Then, 1 g PVA powder was added to the above solution, followed by heating at 80 °C under magnetic stirring. When the solution became clear, the H_3_PO_4_/PVA gel electrolyte was ready. Two aligned CNBs/TPU conductive fibers were twisted together to form an all-solid-state flexible micro-SC, after being coated with the H_3_PO_4_/PVA gel electrolyte. The specific areal capacitance of an electrode can be calculated from the galvanostatic cycling test using the following equation: *C*_A_ = 4*I*Δ*tA*^−1^Δ*V*^−1^, where *I* (A) is the discharge current, Δ*t* (s) is the discharge time, Δ*V* (V) is the potential drop during the discharge process and *A* (cm^−2^) corresponds to the area of the two electrodes (*A* = 2π*DL*, *L* and *D* are the length and diameter of the CNB fibers). The energy density and power density are usually calculated using these equations: *E* = *CV*^2^/8 and *P* = *E*/Δ*t*, where *C* (mF cm^−2^), *V* (V), and Δ*t* (s) are the specific capacitance, operating voltage and discharge time, respectively.

### Detection of ions

Typically, 5 mL of fluorescent CNB solution (0.1 mg mL^−1^) was mixed with 0.5 mL of different concentrations of metal ions. After equilibration for 10 min, fluorescence measurements of these CNB solutions were carried out.

### Characterization

A JEOL JEM-2100 high-resolution transmission electron microscope (HRTEM) was used to observe the microscopic morphologies of CNBs. A NETZSCH STA 449 F3 Jupiter/Nicolet 6700 (TGA/FT-IR) system was used to obtain the thermogravimetric analysis (TGA) curve and the three-dimensional FT-IR profile. Elemental analysis results of the pre-CNBs were obtained using an Elementar Vario EL III. X-ray photoelectron spectroscopy (XPS) spectra of the CNBs were recorded using an ESCALAB 250 XPS. Powder XRD analysis was performed using a SmartLab of Rigaku Corporation. The Raman spectrum of the CNBs was recorded on a Horiba HR 800 Raman system. FT-IR spectra were recorded using a Nicolet 6700 FT-IR spectrometer. The laser confocal fluorescence microscopy (LCFM) images were obtained on a Leica TCS/SP5 system. The time-resolved fluorescence decay curve of the CNBs was also measured on the Leica TCS/SP5 at an excitation wavelength of 405 nm. Photoluminescence (PL) spectra were measured on a Varian Cary Eclipse spectrophotometer. The UV-vis absorption spectrum was recorded on a Lambda 950, PerkinElmer. The electrochemical measurements were performed on a CHI760E electrochemical workstation. The mechanical properties of the CNBs/TPU conductive fibers were investigated using an ETM503C universal strength tester. All the measurements were carried out at room temperature.

## Results and discussion

**Fig. 1:**
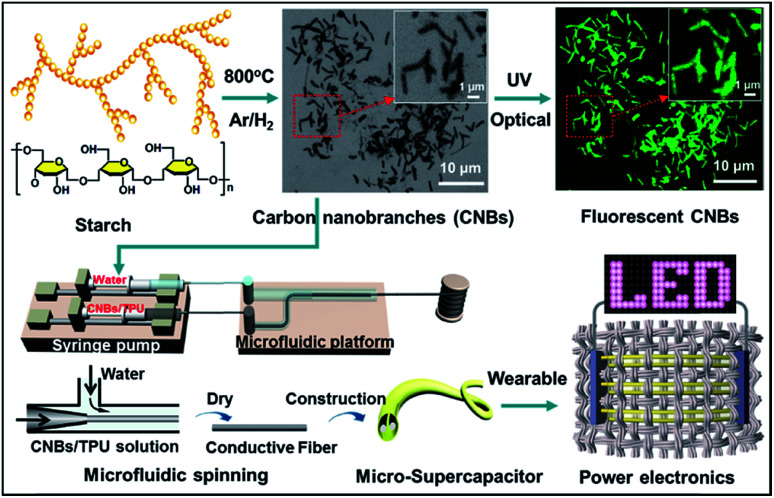
Schematic illustration of the fabrication of CNBs with fluorescence and energy-storage abilities.

Fig. 1 shows the schematic preparation process of CNBs and their versatile applications in chemical sensors and micro-SCs. The pre-CNBs were synthesized *via in situ* pyrolysis of starch in the presence of an Fe-catalyst in an Ar/H_2_ atmosphere at 800 °C. Then, through two-path modification (for details, see the Experimental section), CNBs with photoluminescence (PL) and conductive properties were achieved. In first step, pre-CNBs were treated with concentrated nitric acid, and the obtained sample still exhibited no detectable photoluminescence. In the second path, a bright green PL was observed when the sample was passivated by amine-terminated compounds (TTDDA), as shown in Fig. S1.† It should be pointed out that the precursor and TTDDA are non-fluorescent. Through utilizing the PL properties of the CNBs, a fluorescence sensing probe for selective and sensitive detection of Co^2+^ ions was realized. On the other hand, highly conductive composite CNBs/TPU fibers were fabricated *via* a microfluidic spinning technique. A well dispersed solution of CNBs/TPU was injected into the microchannel using a syringe pump, and coagulated with water through solvent exchange. Then, conductive fibers were formed. An all-solid-state micro-SC was assembled using two pieces of the as-synthesized CNBs/TPU fiber electrodes covered with H_3_PO_4_/PVA gel-type electrolyte. By integrating several micro-SCs in series and sewing them into woven fabric, the wearable micro-SCs powered LEDs successfully.

### Morphologies and structural characterization of CNBs

**Fig. 2:**
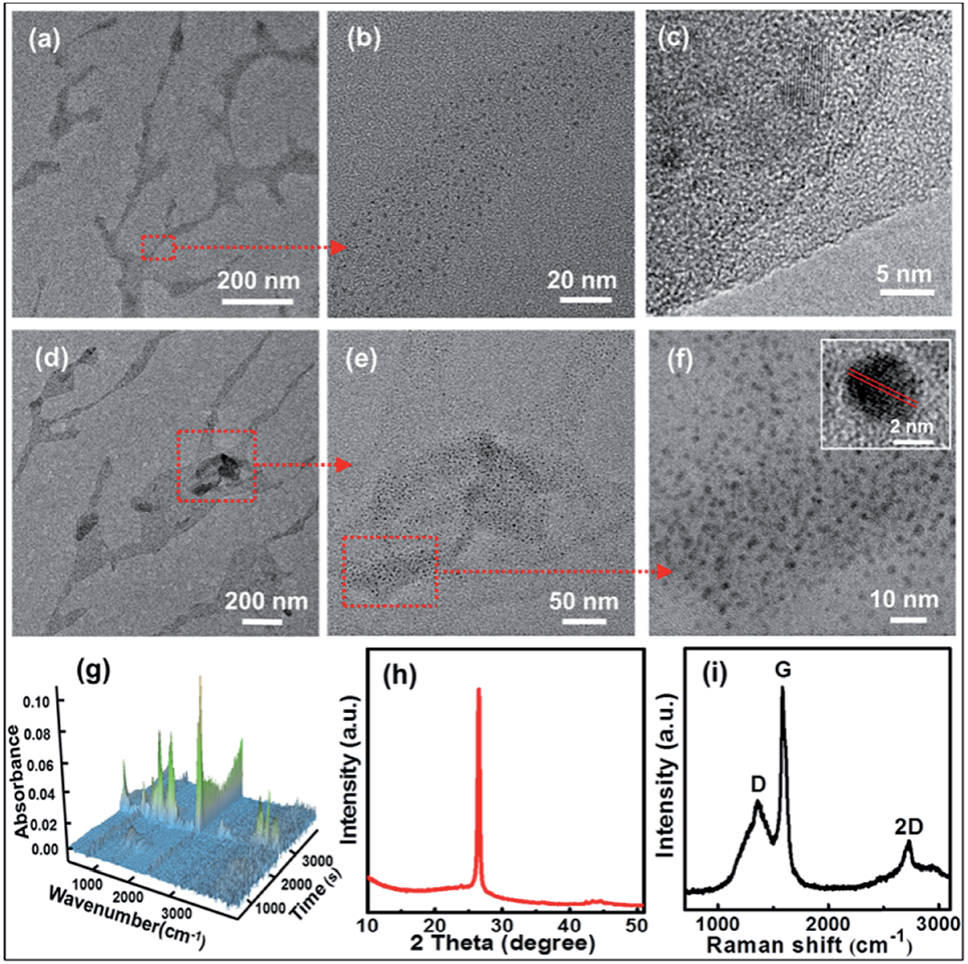
(a and d) TEM images and (b, c, e and f) HRTEM images of the CNBs. (g) 3D FT-IR profile (26 °C at 0 min to 600 °C at 57 min). (h) XRD pattern of CNBs. (i) Raman spectrum of CNBs.

The microscopic morphologies of CNBs were characterized by HRTEM. In Fig. 2a, CNBs present an obviously abundant dendritic structure at low-magnification. The diameters of CNBs are in the range of a few nanometers to hundreds of nanometers, and the lengths are in the range of nanometers to micrometers. It is revealed that the morphology of CNBs exhibits a striking resemblance to the precursor of branched-chain starch.^39^ Notably, uniform and regular CDs are distributed on the CNBs (Fig. 2b). It is clear that several carbon layers serve as the skeleton such that CDs are well-attached on CNBs (Fig. 2c). In order to determine whether the CNBs are covered with CDs everywhere, we further enlarged the branch-like region in the selected area (Fig. 2d). Obviously, the CDs spread everywhere over CNBs (Fig. 2e). Additionally, the darker zone in Fig. 2e demonstrates more CD distribution. Fig. 2f further illustrates that a large amount of CDs is fully distributed within CNBs. The average diameter of CDs is 2.3 nm from Fig. S2.† Furthermore, the CDs show well-resolved lattice fringes in the inset of Fig. 2f. The measured spacing between fringes is 0.182 nm, which is consistent with the (100) facet of graphite.^40^

TGA/FT-IR was performed to clarify the formation of starch-derived CNBs. The starch began to give off huge amounts of CO_2_ (2360 cm^−1^ and 671 cm^−1^) and H_2_O (1508 cm^−1^, 1745 cm^−1^ and 3736 cm^−1^) gases at about 270 °C (24 min), as displayed in Fig. 2g and S3.† Furthermore, the weight loss of 86% of starch confirms a complicated carbonization process from 26 °C to 600 °C (Fig. S4†). Additionally, the corresponding compositions of pre-CNBs are C (93.71 wt%), O (4.04 wt%) and H (2.25 wt%). XPS was used to investigate the surface of pre-CNBs. The full spectrum shows the existence of carbon (C 1s, 284 eV) and oxygen (O 1s, 532 eV), as presented in Fig. S5.† In the high-resolution spectrum (Fig. S6†), the C 1s band can be deconvoluted into three peaks at 284.5 eV, 285.9 eV and 290.3 eV, representing sp^2^/sp^3^ carbons (C–C and C=C), sp^3^ carbons (C–O), and carboxyl carbons (COOH). XRD was performed to analyse the phase structure. In Fig. 2h, the pattern contains a sharp high-intensity peak at 26.5°. This peak represents a lattice spacing of 3.35 Å, which is consistent with the graphite (002) lattice spacing (3.3 Å), implying good crystallization of CNBs.^41^ The Raman spectrum of CNBs exhibits a D band at 1372 cm^−1^, a G band at 1570 cm^−1^ and a 2D band at 2731 cm^−1^, indicating a typical graphite structure with the presence of both sp^2^ and sp^3^ hybrid carbons (Fig. 2i). It is obtained that CNBs show a small increase in the *I*_D_/*I*_G_ ratio to 0.493 by comparison with graphite (*I*_D_/*I*_G_ ratio of 0.365).^42,43^ Therefore, the CNBs may have more disordered carbons than graphite. To demonstrate that TTDDA molecules attach to the surface of pre-CNBs, the FT-IR spectra are compared. As presented in Fig. S7,† the absorption peaks of CNBs before passivation (black line) around 1720 cm^−1^ belong to C=O stretching vibrations in the carboxylic acid group (–COOH), indicating the presence of –COOH. However, after the passivation of CNBs (red line), the absorption peak at 1720 cm^−1^ almost disappeared, and other strong absorption peaks at 675 cm^−1^, 1058 cm^−1^ and 1560 cm^−1^ emerged, representing N–H wagging vibration, C–N stretching vibration and amide-II (C–N–H) coupled vibration, respectively. These results reveal that the TTDDA is successfully attached to the pre-CNB surface by acylation reaction (Fig. S1†).^44,45^ A possible explanation for the generation of the dendritic CNBs from starch is that powerful energy resulted in carbonization of the precursor, simultaneously broke long carbon chains into short carbon chains and generated abundant crystal defects in the presence of the Fe-catalyst during the high temperature period. In the process of nitrification, molecules on the surface of the pre-CNBs might change into individual carbon nanoparticles, and the internal molecules of pre-CNBs might play the part of a dendritic skeleton for CNBs.

### Fluorescent properties and ion detection of CNBs

**Fig. 3:**
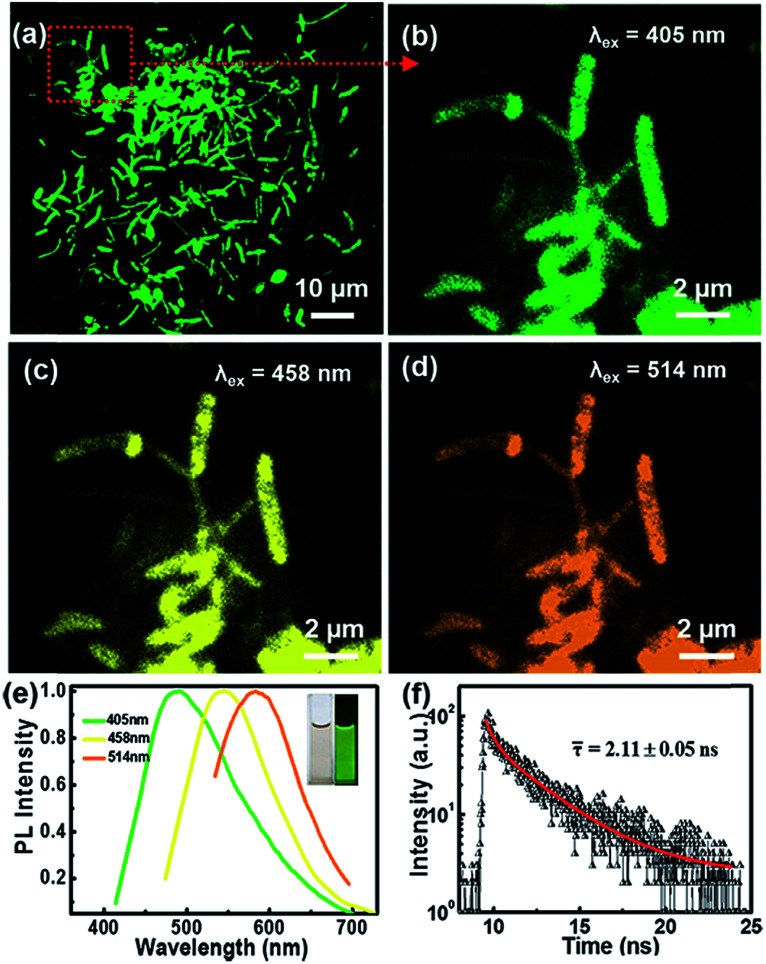
(a) LCFM image of the full view of CNBs (*λ*_ex_ = 405 nm). The enlarged LCFM images of CNBs were obtained under excitation at (b) 405 nm, (c) 458 nm and (d) 514 nm. (e) PL emission spectra of CNB aqueous solution. Inset: photos of the aqueous solution under sunlight and a UV lamp (365 nm). (f) PL decay lifetime spectrum and fitting curve (red line) of CNBs (*λ*_ex_ = 405 nm).

The microstructures and PL properties of CNBs were investigated using laser confocal fluorescence microscopy (LCFM). As the low-magnification LCFM images of CNBs show in Fig. 3a and S8,† lots of branch-shaped lines with bright green fluorescence are clearly observed under UV light with a uniform dispersion. The diameters of CNBs are in range of tens to hundreds of nanometers, which are relatively wider than those in HRTEM images. A halo of green fluorescence around CNBs resulted in the wider diameters determined by LCFM. Fig. 3b shows a high-magnification LCFM image of CNBs, which further demonstrates similar branch-like microstructures of CNBs in Fig. 2a and d. Meanwhile, homogeneous yellow and orange PL emissions can also be seen when they were excited at 458 nm and 514 nm (Fig. 3c and d). The optical properties of colloidal CNBs were studied utilizing the PL emission spectrum. As shown in Fig. 3e and S9,† the emission spectra of CNBs exhibit excitation-dependent PL behavior. As the excitation wavelength increases from 380 nm to 540 nm, the emission peak shifts from 504 nm to 590 nm. The CNB aqueous solution displays a bright green color under a UV lamp (365 nm) in Fig. 3e. In addition, the average lifetime of CNBs is calculated to be 2.11 ± 0.05 ns (Fig. 3f). The UV-vis absorption spectrum of CNBs in Fig. S10† shows an absorption peak at about 315 nm. It has been reported that CDs behave as a kind of indirect band gap nanomaterial.^46^ The indirect band gap *E*_g_ of CNBs was estimated to be 2.20 eV in Fig. S11† on the basis of the UV-vis spectrum, which indicates nanoscale CNBs with quantum size effects.^47^ Furthermore, the as-prepared CNBs show no obvious deterioration in PL intensity after being stored for 6 months (Fig. S12†), revealing excellent fluorescent stability.

**Fig. 4:**
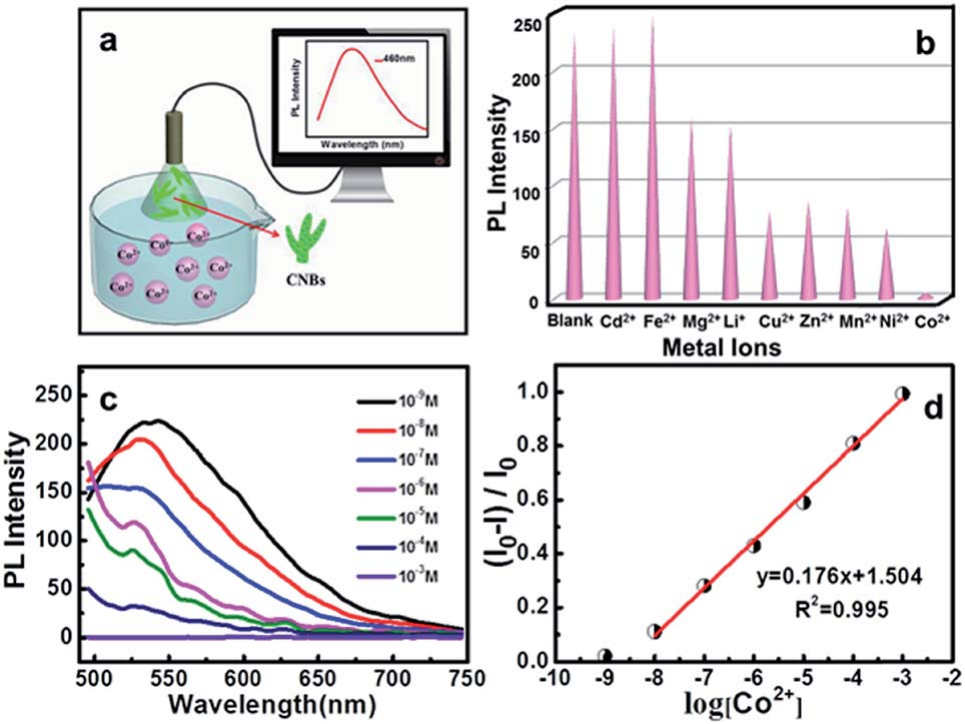
(a) Schematic diagram depicting the sensing application of CNBs for detection of Co^2+^. (b) Comparison of PL intensities of CNB solution in the absence and presence of different ions (*λ*_ex_ = 460 nm, [M^*n*+^] = 10^−3^ mol L^−1^). (c) Effect of different concentrations of Co^2+^ on the fluorescence of CNB solution (from top to bottom: 10^−9^, 10^−8^, 10^−7^, 10^−6^, 10^−5^, 10^−4^, and 10^−3^ M). (d) The calibration plot of the values of (*I*_0_ − *I*)/*I*_0_ of CNBs *versus* the concentrations of Co^2+^ in the range of 10^−9^ to 10^−3^ M. *I* and *I*_0_ are the PL intensities of CNBs at 460 nm in the presence and absence of Co^2+^, respectively.

Recently, fluorescent carbon nanosensors have been rapidly developed. Sensing application of CNBs towards metal ions was examined here. Fig. 4a displays the schematic diagram of the sensing application of CNBs towards Co^2+^ in water. Initially, to investigate the selectivity of the sensing property, the PL intensity in the presence of different metal ions was compared, including Cd^2+^, Fe^2+^, Mg^2+^, Li^+^, Cu^2+^, Zn^2+^, Mn^2+^, Ni^2+^ and Co^2+^, as seen in Fig. 4b. Cobalt ions displayed a strong fluorescence quenching effect, compared with other cations. The emission intensity of CNBs was nearly unaffected by Cd^2+^ and Fe^2+^, and partly quenched by Mg^2+^, Li^+^, Cu^2+^, Zn^2+^, Mn^2+^ and Ni^2+^. It is revealed that the as-prepared CNBs show PL selectivity in response to Co^2+^ ions, which has rarely been reported in the literature.^48–50^ The reason for fluorescence quenching may be attributed to electron or energy transfer through Co^2+^ ions with an unfilled orbit.^51,52^ On the other hand, to investigate the sensitivity of the CNBs towards Co^2+^, the PL emission spectra of CNB dispersion were obtained at different Co^2+^ concentrations varying from 10^−3^ M to 10^−9^ M (Fig. 4c). It is found that the fluorescence intensity of CNBs gradually decreased with increasing Co^2+^ concentration. The normalized calibration plot of (*I*_0_ – *I*)/*I*_0_ of CNBs against Co^2+^ is shown in Fig. 4d. The linear concentration range for Co^2+^ covers 10^−8^ to 10^−3^ M with a regression coefficient, *R*^2^ = 0.995. The detection limit defined as the concentration of Co^2+^ giving a signal equivalent to a blank signal plus three times the standard deviation of the blank was calculated to be 2.85 nM.^53,54^ It is worth noting that the CNBs exhibit superior selectivity and sensitivity towards cobalt ions, which can be exceptionally designed as a fluorescent probe in environmental and biological research.

### Electrochemical performance and application of CNBs/TPU fiber-based micro-SCs

**Fig. 5:**
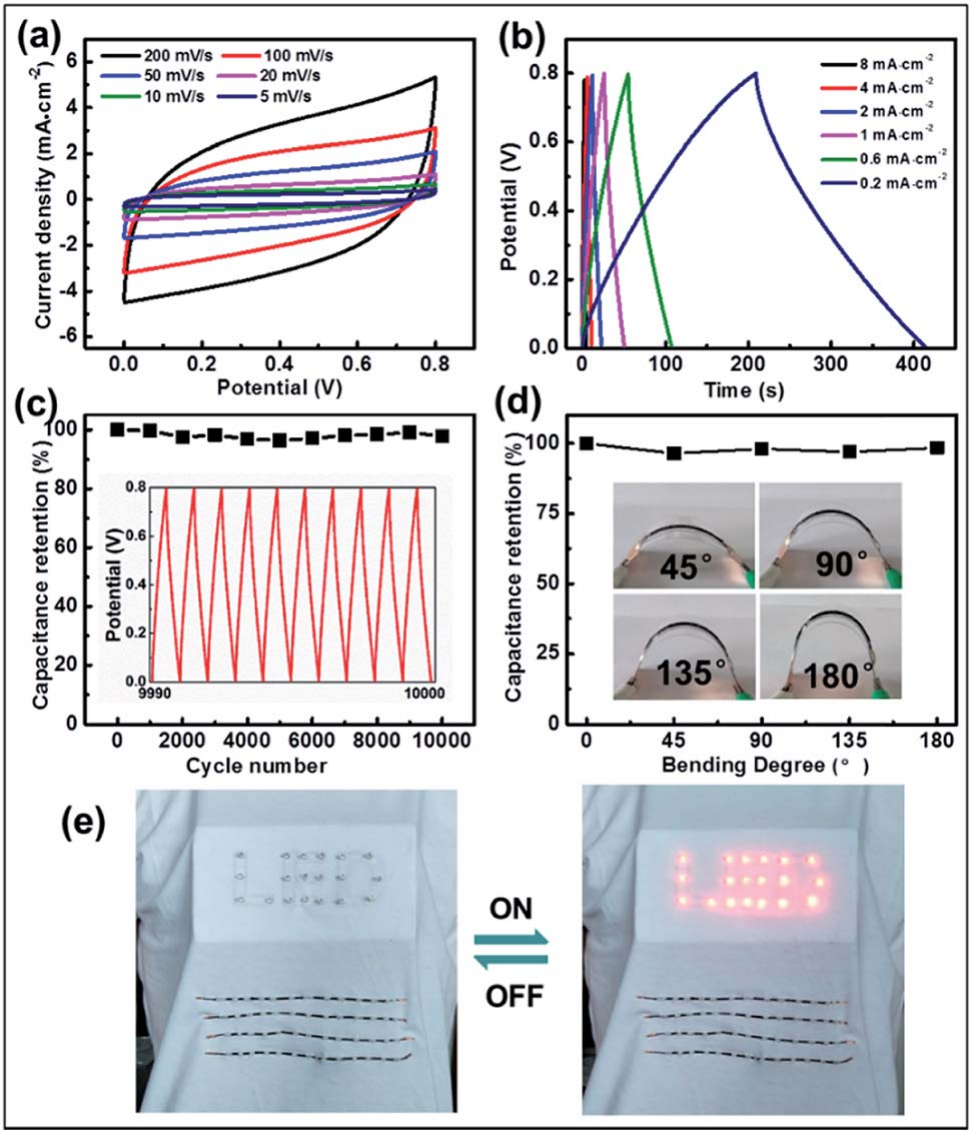
(a) CV curves of micro-SCs based on CNB fibers in H_3_PO_4_/PVA solid-state electrolyte, with scan rates increased from 5 to 10, 20, 50, 100 and 200 mV s^−1^. (b) GCD curves of micro-SCs, with current density increased from 0.2 to 0.6, 1, 2, 4 and 8 mA cm^−2^. (c) The cycling stability of micro-SCs at a voltage of 0.8 V at a current density of 0.4 mA cm^−2^; the inset: GCD curves after 10 000 cycles between 0 and 0.8 V. (d) Capacitance retention of the CNB micro-SCs at different bending states at a current density of 0.4 mA cm^−2^. (e) Four CNB micro-SCs were connected in series into woven fabric and powered 19 LEDs.

To further exploit their application, effective micro-SCs based on a CNBs/TPU composite fiber electrode were significantly constructed where the fibers were prepared *via* a microfluidic spinning technique. Fig. S13 and S14† illustrate the electrical and mechanical properties of CNBs/TPU fibers. Significantly, excellent electrical conductivity (1443 S m^−1^), tensile strength (15.9 MPa), and mechanical elongation (14.7%) of fibers were achieved. Owing to the continuous microfluidic spinning process, CNBs/TPU fibers of more than 1 m in length can be produced (Fig. S15a†) with a diameter of 320 μm (Fig. S15b and c†). Two aligned CNBs/TPU fibers were twisted together and further covered with H_3_PO_4_/PVA gel electrolyte of about 65 μm (Fig. S16†) to construct micro-SCs. Electrochemical performances of micro-SCs were tested using cyclic voltammetry (CV) and galvanostatic charge/discharge (GCD) measurements. The CV curves of micro-SCs exhibit a slightly inclined rectangular shape when increasing the scan rate from 5 to 200 mV s^−1^ as shown in Fig. 5a, indicating the presence of electric double layer capacitance. Additionally, the GCD curves show a basically symmetric triangular shape in Fig. 5b, indicating good reversibility in charge and discharge cycles. The specific areal capacitance was estimated using galvanostatic discharge curves. As shown in Fig. S17,† the specific areal capacitance of micro-SCs decreased as the current density increased. A largest specific capacitance of 201.4 mF cm^−2^ at a current density of 0.2 mA cm^−2^ can be achieved, which is higher than the values of other types of carbon nanomaterials^55,56^ (mesoporous carbon/CNTs 39.7 mF cm^−2^ and graphene/CNTs 177 mF cm^−2^). In addition, the CNB micro-SCs could still maintain a high capacitance of 81.2 mF cm^−2^ at a current density of 8 mA cm^−2^, implying excellent energy-storage performance. Most significantly, it is the large specific surface area of CNBs that makes a great contribution to the specific capacitance of micro-SCs. Meanwhile, energy density and power density are important performance parameters for micro-SCs. As shown in Fig. S18,† the micro-SCs show the energy densities of 1.8–4.5 μW h cm^−2^ at the power densities of 3.2–0.08 mW cm^−2^. To our knowledge, the maximum energy density of our micro-SCs can be comparable with that of other reported fiber-based micro-SCs, such as CNT@Co_3_O_4_ (1.03 μW h cm^−2^),^57^ rGO/Ni (1.60 μW h cm^−2^),^58^ PANI/MCNTs-rGO/TPU (3.45 μW h cm^−2^),^59^ and rGO-CNT@CMC (3.84 μW h cm^−2^).^56^ Furthermore, the cycling stability was performed by a continuous charging and discharging process at a voltage of 0 to 0.8 V at a current density of 0.4 mA cm^−2^. The micro-SCs displayed a capacitance retention of 98% after 10 000 cycles, demonstrating their impressive electrochemical stability (Fig. 5c). Moreover, the micro-SC was subjected to a bending test at bending angles of 45°, 90°, 135° and 180°, respectively (Fig. 5d). Negligible capacitance deterioration was observed when undergoing various bending deformation, indicating the high flexibility of micro-SCs which is desirable for wearable applications. Several micro-SCs were assembled in series and in parallel to increase their cell voltage or capacity for practical applications. The operating voltage window could be tripled from 0.8 V to 2.4 V by connecting three devices in series, as seen in Fig. S19.† Meanwhile, the output current and discharge time could also be tripled by connecting three devices in parallel, as shown in Fig. S20.† Through such an integration of four micro-SCs in series and sewing into woven fabric, 19 LEDs are successfully lit (Fig. 5e).

## Conclusions

In summary, we have developed a facile approach for the fabrication of CNBs by pyrolysis of starch, whereby the as-prepared CNBs are totally covered with abundant CDs. By surface modification treatment, the CNBs display green PL under UV excitation. Additionally, the CNBs exhibit an excellent crystalline structure and superior selectivity and sensitivity as potential fluorescent probes for Co^2+^ detection. Furthermore, the highly conductive CNBs/TPU fibers were prepared by using a customized microfluidic spinning instrument. The fiber-based micro-SCs show excellent electrochemical performance, including large specific capacitance (201.4 mF cm^−2^), high energy densities of 1.8–4.5 μW h cm^−2^ at the power densities of 3.2–0.08 mW cm^−2^, great electrochemical stability after 10 000 cycles and high flexibility. Through integrating micro-SCs into woven fabric, 19 LEDs were successfully lit. Considering these amazing achievements, this approach will not only offer an avenue to design the multifunctional 1D nanomaterials, but also greatly promote the development of nanomaterial applications in sensors and energy storage devices.

## Conflicts of interest

The authors declare no competing financial interests.

## Supplementary Material

NA-001-C9NA00181F-s001

## References

[cit1] Baughman R. H., Zakhidov A. A., de Heer W. A. (2002). Science.

[cit2] Iijima S. (1991). Nature.

[cit3] Shen Y., Yan L., Song H., Yang J., Yang G., Chen X., Zhou J., Yu Z.-Z., Yang S. (2012). Angew. Chem., Int. Ed..

[cit4] De Jong K. P., Geus J. W. (2000). Catal. Rev.: Sci. Eng..

[cit5] Endo M., Kroto H. W. (1992). J. Phys. Chem..

[cit6] Shen L., Zeng M., Yang S.-W., Zhang C., Wang X., Feng Y. (2010). J. Am. Chem. Soc..

[cit7] Baughman R. H. (2006). Science.

[cit8] Tang Y. H., Wang N., Zhang Y. F., Lee C. S., Bello I., Lee S. T. (1999). Appl. Phys. Lett..

[cit9] Orikasa H., Akahane T., Okada M., Tong Y., Ozakib J., Kyotania T. (2009). J. Mater. Chem..

[cit10] Sun L., Gong J., Zhu D., Zhu Z., He S. (2004). Adv. Mater..

[cit11] Chen L., Hernandez Y., Feng X., Mullen K. (2012). Angew. Chem., Int. Ed..

[cit12] Yu S.-S., Zheng W.-T. (2010). Nanoscale.

[cit13] Shang N., Papakonstantinou P., Wang P., Zakharov A., Palnitkar U., Lin I., Chu M., Stamboulis A. (2009). ACS Nano.

[cit14] Li J., Papadopoulos C., Xu J. (1999). Nature.

[cit15] Wu C. C., Liu C. H., Zhong Z. (2010). Nano Lett..

[cit16] Cao X., He Q., Shi W., Li B., Zeng Z., Shi Y., Yan Q., Zhang H. (2011). Small.

[cit17] Huang B., Li Z., Liu Z., Zhou G., Hao S., Wu J., Gu B.-L., Duan W. (2008). J. Phys. Chem. C.

[cit18] Yang Z., Liu M., Zhang C., Tjiu W. W., Liu T., Peng H. (2013). Angew. Chem., Int. Ed..

[cit19] Zheng X., Chen H., Li Q., Yang Y., Wei Z., Bai Y., Qiu Y., Zhou D., Wong K. S., Yang S. (2017). Nano Lett..

[cit20] Cao Y., Xiao L., Sushko M. L., Wang W., Schwenzer B., Xiao J., Nie Z., Saraf L. V., Yang Z., Liu J. (2012). Nano Lett..

[cit21] Li Z., Zhang J., Lou X. W. (2015). Angew. Chem., Int. Ed..

[cit22] Wang R., Yan X. (2014). Sci. Rep..

[cit23] Liu H., Jin L.-H., He P., Wang C., Xia Y. (2009). Chem. Commun..

[cit24] Liu C., Fan Y. Y., Liu M., Cong H. T., Cheng H. M., Dresselhaus M. S. (1999). Science.

[cit25] Mananghaya M., Yu D., Santos G. N., Rodulfo E. (2016). Sci. Rep..

[cit26] Ren W., Wang Y., Tan Q., Zhong Z., Su F. (2016). J. Power Sources.

[cit27] Xia X., Chao D., Zhang Y., Zhan J., Zhong Y., Wang X., Wang Y., Shen Z. X., Tu J., Fan H. J. (2016). Small.

[cit28] Meyer J. C., Girit C. O., Crommie M. F., Zettl A. (2008). Nature.

[cit29] Pachfule P., Shinde D., Majumder M., Xu Q. (2016). Nat. Chem..

[cit30] Bai J., Duan X., Huang Y. (2009). Nano Lett..

[cit31] Nakao H., Tokonami S., Yamamoto Y., Shiigid H., Takeda Y. (2014). Chem. Commun..

[cit32] Lin J., Peng Z., Xiang C., Ruan G., Yan Z., Natelson D., Tour J. M. (2013). ACS Nano.

[cit33] Zhao Y., Zhao F., Wang X., Xu C., Zhang Z., Shi G., Qu L. (2014). Angew. Chem., Int. Ed..

[cit34] Xia X., Zeng Z., Li X., Zhang Y., Tu J., Fan N. C., Zhang H., Fan H. J. (2013). Nanoscale.

[cit35] Ham J., Park J. Y., Dong W. J., Jung G. H., Yu H. K., Lee J.-L. (2016). Appl. Phys. Lett..

[cit36] Son K.-S., Lee D. H., Choung J.-W., Pyun Y. B., Il Park W., Song T., Paik U. (2008). J. Mater. Res..

[cit37] Kou X., Sun Z., Yang Z., Chen H., Wang J. (2009). Langmuir.

[cit38] Zhou H., Tang Y., Zhai J., Wang S., Tang Z., Jiang L. (2009). Sensors.

[cit39] Jane J., Chen Y. Y., Lee L. F., McPherson A. E., Wong K. S., Radosavljevic M., Kasemsuwan T. (1999). Cereal Chem..

[cit40] Baker S. N., Baker G. A. (2010). Angew. Chem., Int. Ed..

[cit41] Bourlinos B., Stassinopoulos A., Anglos D., Zboril R., Georgakilas V., Giannelis E. P. (2008). Chem. Mater..

[cit42] Zhou J., Booker C., Li R., Zhou X., Sham T.-K., Sun X., Ding Z. (2007). J. Am. Chem. Soc..

[cit43] Li H., Kang Z., Liu Y., Lee S.-T. (2012). J. Mater. Chem..

[cit44] Sun Y.-P., Zhou B., Lin Y., Wang W., Fernando K. A. S., Pathak P., Meziani M. J., Harruff B. A., Wang X., Wang H., Luo P. G., Yang H., Kose M. E., Chen B., Veca L. M., Xie S.-Y. (2006). J. Am. Chem. Soc..

[cit45] Peng H., Travas-Sejdic J. (2009). Chem. Mater..

[cit46] Tian L., Ghosh D., Chen W., Pradhan S., Chang X., Chen S. (2009). Chem. Mater..

[cit47] Tsunekawa S., Fukuda T. (2000). J. Appl. Phys..

[cit48] Liu S., Tian J. Q., Wang L., Zhang Y. W., Qin X. Y., Luo Y. L., Asiri A. M., Al-Youbi A. O., Sun X. P. (2012). Adv. Mater..

[cit49] Zhou L., Lin Y., Huang Z., Ren J., Qu X. (2012). Chem. Commun..

[cit50] Chan Y.-H., Jin Y., Wu C., Chiu D. T. (2011). Chem. Commun..

[cit51] Li X., Zhang S., Kulinich S. A., Liu Y., Zeng H. (2014). Sci. Rep..

[cit52] Rahimi Y., Goulding A., Shrestha S., Mirpuri S., Deo S. K. (2008). Biochem. Biophys. Res. Commun..

[cit53] Ertekin K., Oter O., Ture M., Denizalti S., Cetinkaya E. (2010). J. Fluoresc..

[cit54] Yusof N. A., Rahman W. A., Kadir W. A. (2009). Spectrochim. Acta, Part A.

[cit55] Ren J., Bai W., Guan G., Zhang Y., Peng H. (2013). Adv. Mater..

[cit56] Kou L., Huang T., Zheng B., Han Y., Zhao X., Gopalsamy K., Sun H., Gao C. (2014). Nat. Commun..

[cit57] Su F., Lv X., Miao M. (2015). Small.

[cit58] Pu X., Li L., Liu M., Jiang C., Du C., Zhao Z., Hu W., Wang Z. L. (2016). Adv. Mater..

[cit59] Tong Y.-L., Xu B., Du X.-F., Cheng H.-Y., Wang C.-F., Wu G., Chen S. (2018). Macromol. Mater. Eng..

