# Supplementary material for: Facile synthesis of carbon nanobranches towards cobalt ion sensing and high-performance micro-supercapacitors
Source: Nanoscale Adv. 2019 Jul 19;1(9):3614–20. doi: 10.1039/c9na00181f (PMC9417816; doi:10.1039/c9na00181f)
Supplement: NA-001-C9NA00181F-s001 [file NA-001-C9NA00181F-s001.pdf]

Electronic Supplementary Material (ESI) for Nanoscale Advances.
This journal is © The Royal Society of Chemistry 2019

Supporting Information

Facile Synthesis of Carbon Nanobranches towards Cobalt Ion Sensing and High-Performance Micro-Supercapacitors

Qiao-Ling Chen, Xingjiang Wu, Hengyang Cheng, Qing Li and Su Chen**

State Key Laboratory of Materials-Oriented Chemical Engineering and College of Chemical Engineering, Nanjing Tech University (former Nanjing University of Technology), Nanjing 210009, P. R. China.

*Correspondent author: Tel: 86-25-83172258, Fax: 86-25-83172258, E-mail: chensu@njtech.edu.cn, liqing1128@njtech.edu.cn

Supplementary Figures

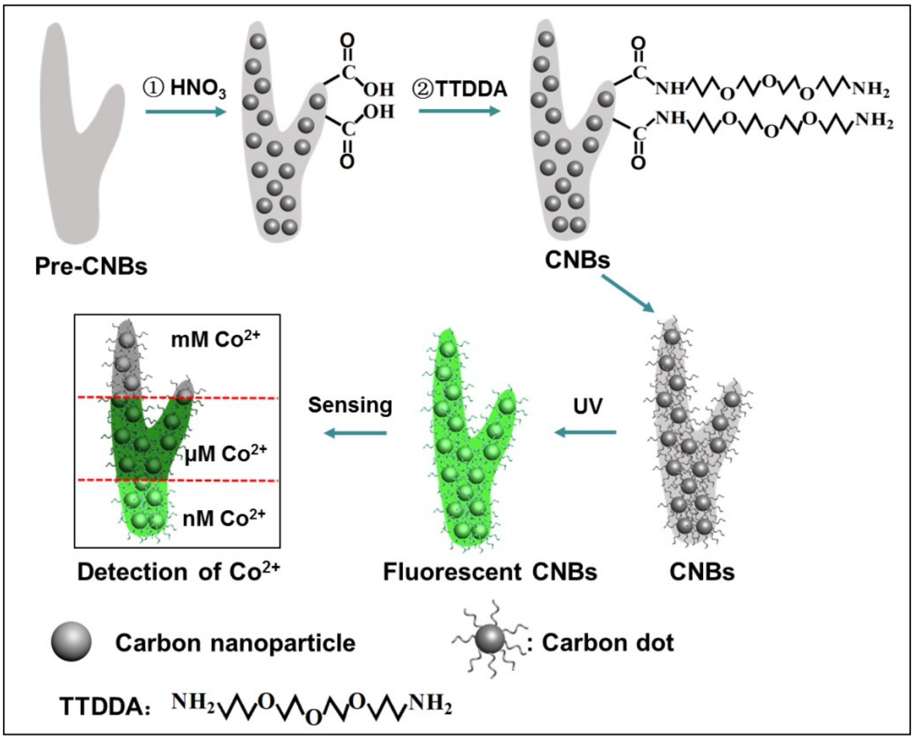

Figure S1. The chemical reaction principle of the two-path modification of CNBs.

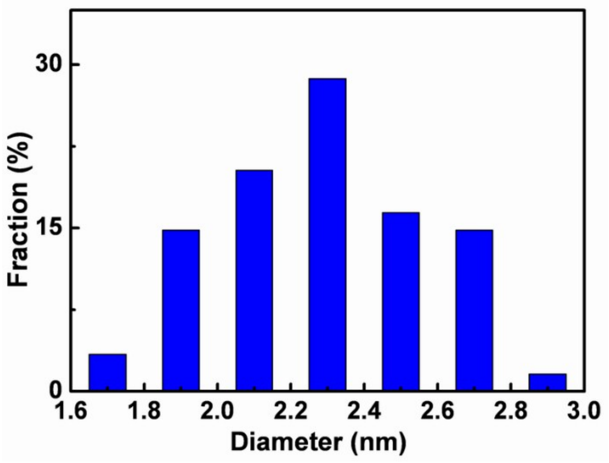

Figure S2. Particle size distribution of CDs.

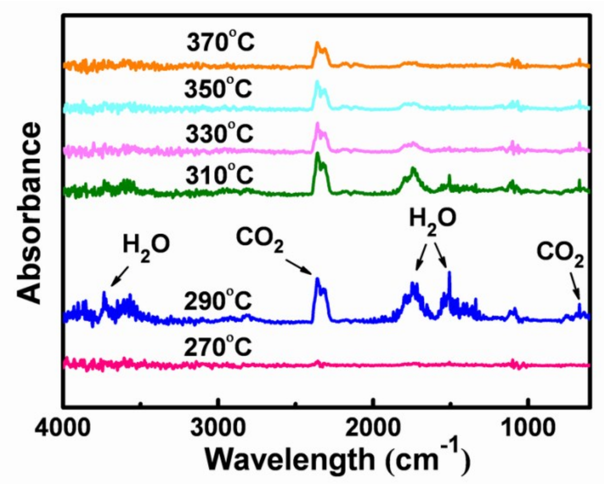

Figure S3. Evolutionary FT-IR spectra (270 ^{o}C to 370 ^{o}C) of gas produced from starch combustion.

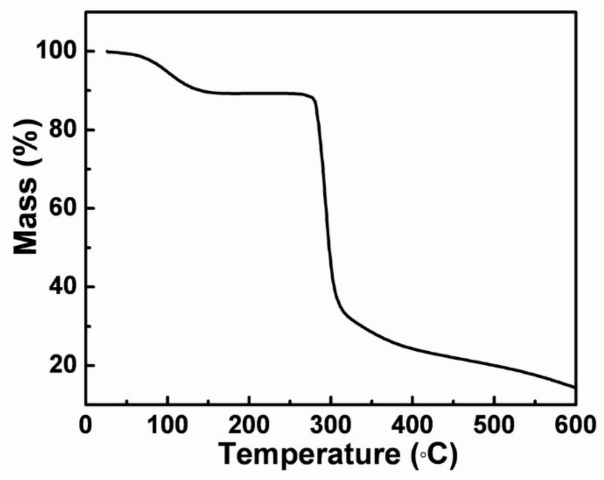

Figure S4. TGA curve of starch from 26 ^{o}C to 600 ^{o}C.

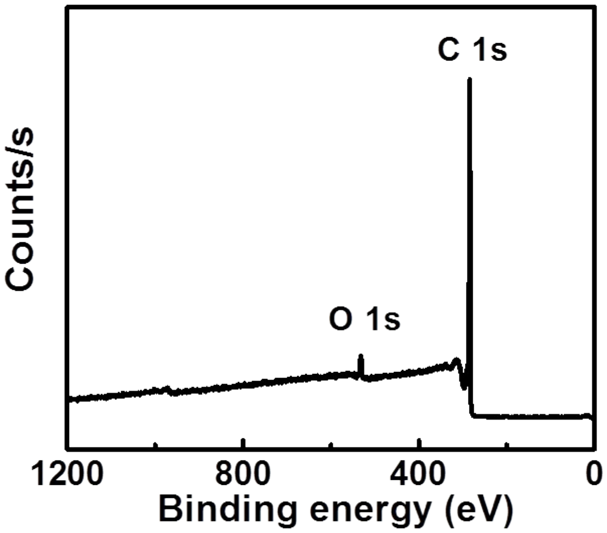

Figure S5. XPS spectrum of the CNBs.

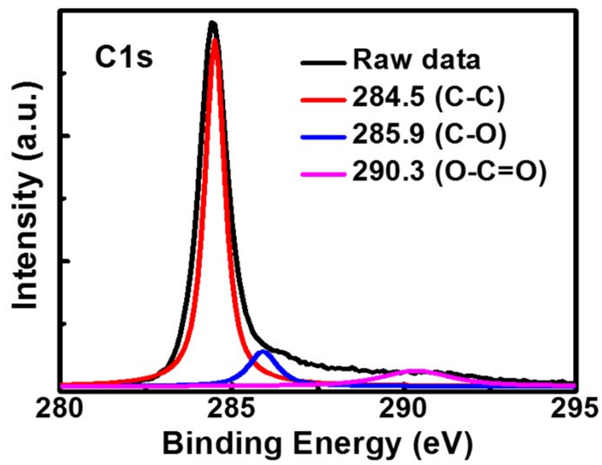

Figure S6. High resolution XPS spectrum of C 1s peak of the CNBs.

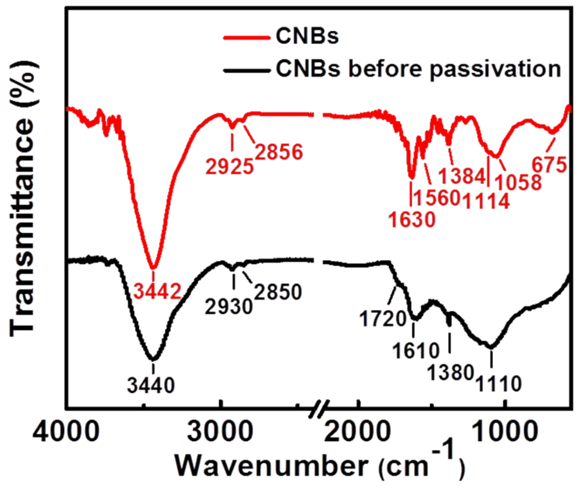

Figure S7. FT-IR spectra of CNBs and CNBs before passivation.

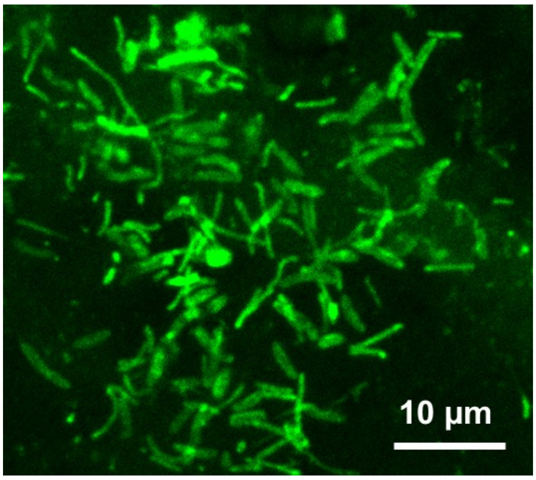

Figure S8. Laser confocal fluorescence microscopy (LCFM) image of CNBs at excitation wavelengths of 405 nm.

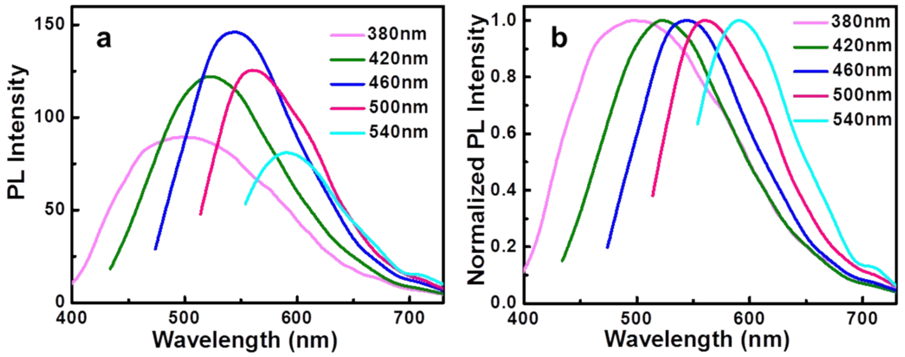

Figure S9. (a) PL emission spectra and (b) normalized PL emission spectra of CNBs in aqueous solution at excitation wavelengths starting from 380 nm to 540 nm in 40 nm increments.

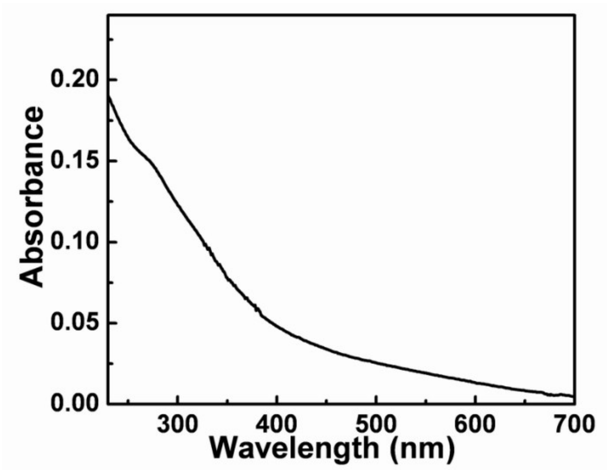

Figure S10. UV-vis absorption spectrum of CNBs aqueous solution.

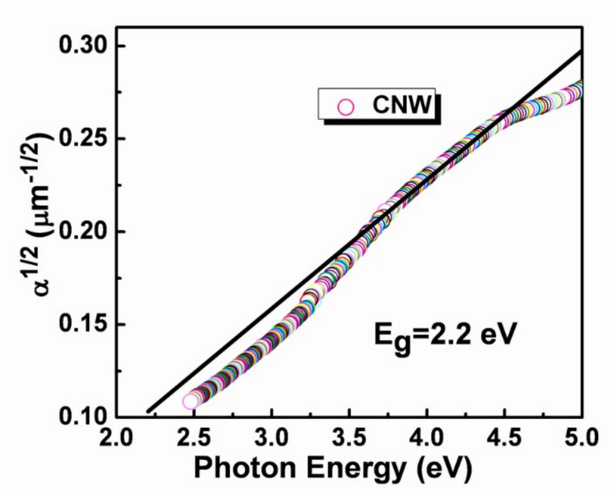

Figure S11. Plot of $\alpha^{1/2}$ vs E_{phot} for indirect transitions, where α is absorption coefficient and E_{phot} is photon energy. Band gaps E_{g} are obtained by extrapolation to α =0.

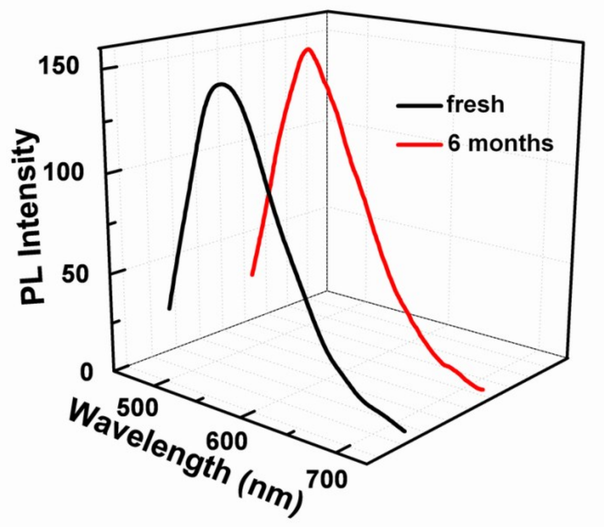

Figure S12. PL spectra of fresh and six-month-stored solutions of CNBs (λ_{ex} = 460 nm).

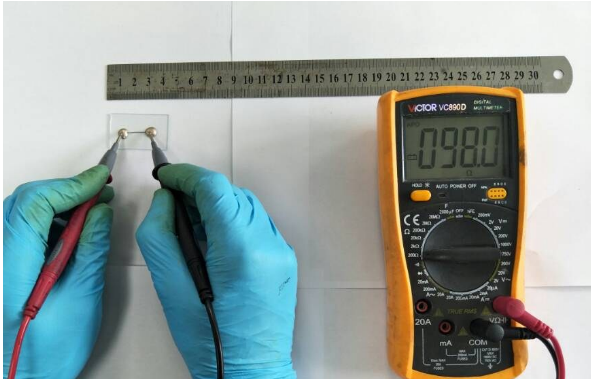

Figure S13. The photograph shows the electrical conductivity of a CNBs/TPU fiber.

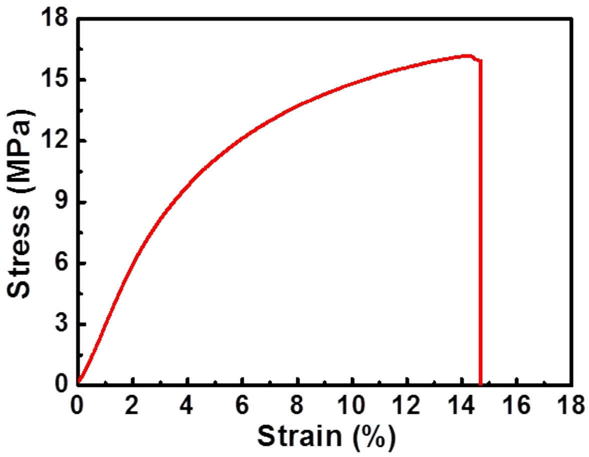

Figure S14. Typical stress-strain curve of a CNBs/TPU fiber.

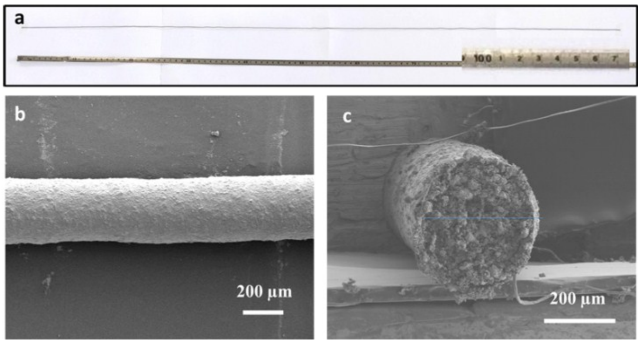

Figure S15. (a) Optical photograph of a long CNBs/TPU fiber. SEM images of side (b) and cross-sectional (c) view of a CNBs/TPU fiber.

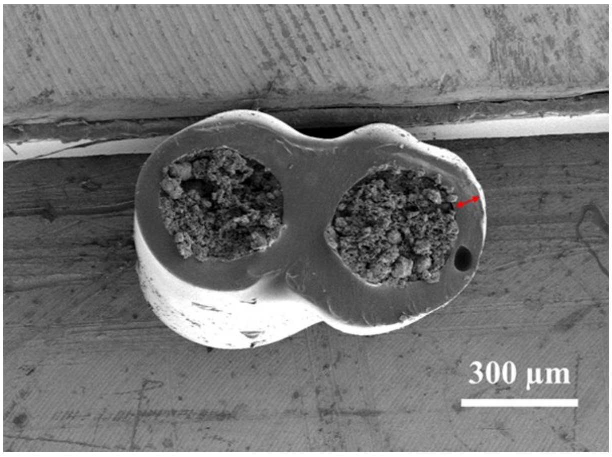

Figure S16. SEM image of cross-sectional view of a CNBs/TPU fiber-based micro-SCs. Red arrow area is H_3PO_4/PVA gel electrolyte.

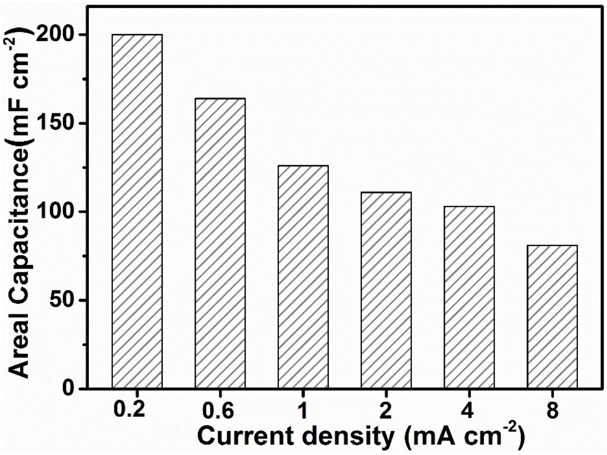

Figure S17. The calculated specific capacitances under different current densities.

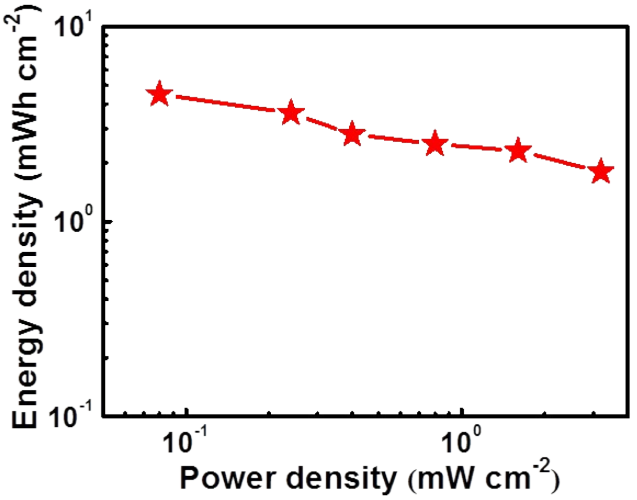

Figure S18. Corresponding energy density and power density of the CNBs/TPU fiber-based micro-SCs.

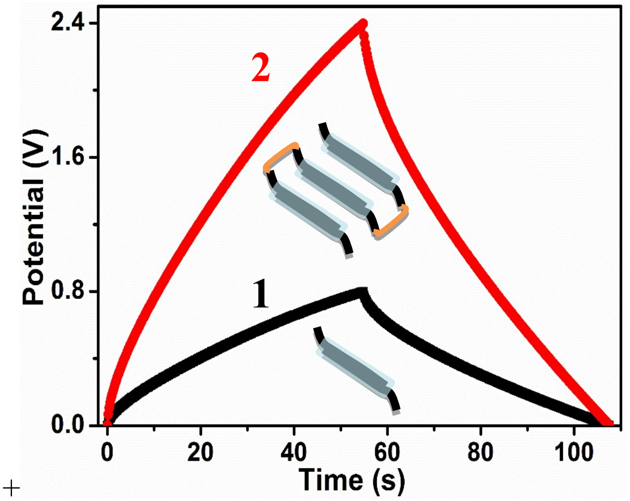

Figure S19. Three micro-SCs connected in series.

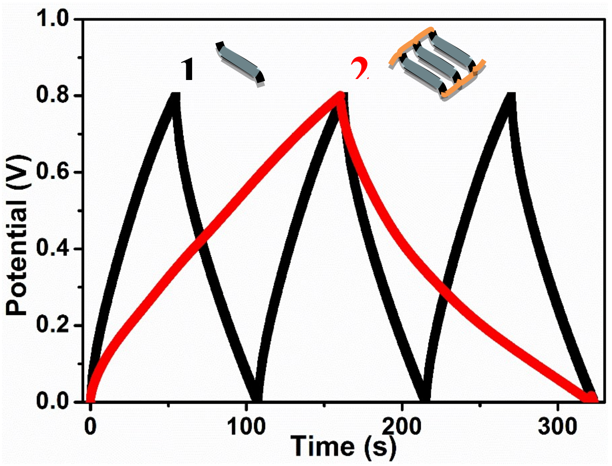

Figure S20. Three micro-SCs connected in parallel.
